# Intraperitoneal Levonorgestrel-Releasing Intrauterine Device in a Patient With a Kidney Stone: A Case Report

**DOI:** 10.7759/cureus.95251

**Published:** 2025-10-23

**Authors:** Austin Goldsamt, Jeffrey L Lord, Nathan D Alessio

**Affiliations:** 1 Department of Surgery, Boston Medical Center Health System, Boston, USA; 2 Department of Surgery, Holy Family Hospital, Methuen, USA

**Keywords:** ectopic intrauterine device, intraperitoneal, intrauterine device, laparoscopy, uterine perforation

## Abstract

This report presents a rare case of a previously placed intrauterine device (IUD) subsequently found freely floating in the peritoneal cavity in a patient presenting with a kidney stone. A woman in her early 30s with a history of levonorgestrel IUD placement four years prior presented to the emergency department with sudden-onset, sharp, severe left lower quadrant pain. A CT scan demonstrated a 5 mm obstructing ureteral stone in the distal left ureter as well as an IUD in the inferior peritoneal cavity with no free air or bowel injury. This case, in the context of current literature surrounding migrated and perforated IUDs, is evaluated and presented in the following report. The device was successfully retrieved laparoscopically without incident. Intraoperative inspection showed no uterine perforation at the time of laparoscopy, no adhesions, and no involvement of adjacent structures or organs. Postoperative follow-up was conducted to assess recovery and symptom resolution. Intraperitoneal migration of IUDs, although rare, can occur and may remain asymptomatic for several years. Migration may be complicated by adhesions, perforation, and infection. However, ectopic IUDs may alternatively be free-floating in the peritoneal cavity without complications. Laparoscopic retrieval is safe and effective, and this can be considered the preferred method of retrieval in most cases.

## Introduction

There are numerous methods of contraception for females. These include surgical methods such as tubal ligation. Other examples include spermicides, vaginal sponges, male condom use, withdrawal during intercourse, contraceptive rings, oral contraceptive pills, contraceptive injections, and subcutaneous implants such as the Nexplanon [1]. Among these is the intrauterine device (IUD), which provides long-acting, reversible contraception. It is also safe and cost-effective, rendering it the most utilized method of contraception [2]. IUDs work by creating a sterile foreign body reaction, creating a hostile environment for sperm and the fertilization process. In the case of a progestin-containing IUD, thickening the cervical mucus hinders sperm entry [3]. IUDs are very safe; complications with IUDs occur in fewer than 1% of women [3]. Complications may occur at the time of IUD insertion or later [3]. This case involves a migrated IUD, which refers to the movement of the device from its normal position in the uterine cavity to another location (within or outside of the uterus). In this patient, the IUD was found free floating in the peritoneal cavity (the potential space between the visceral and parietal peritoneum). There were no other complications or involved structures, and the IUD was successfully retrieved with laparoscopy.

## Case presentation

**Figure 1:**
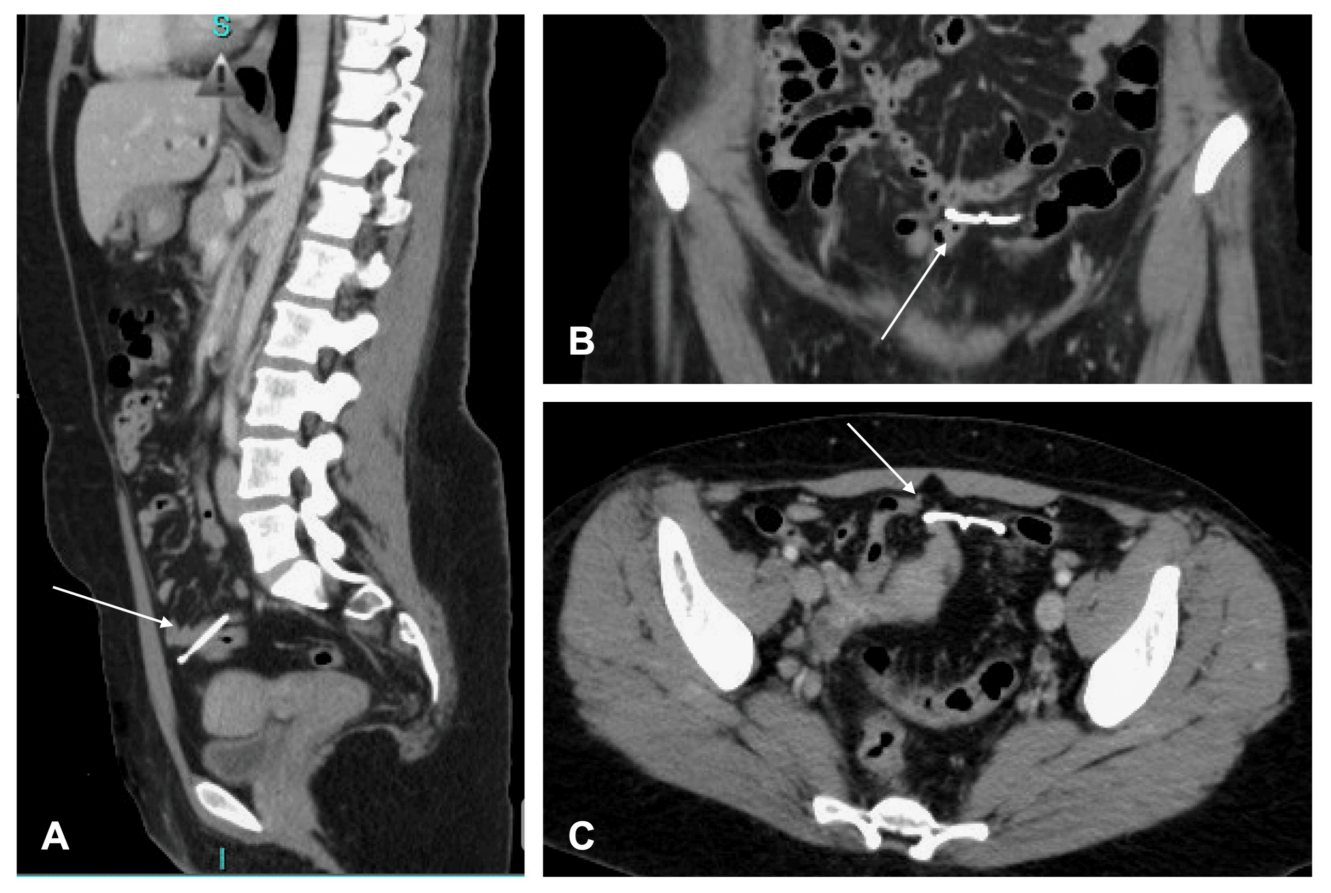
CT scan of the abdomen/pelvis, sagittal view (A), coronal view (B), and axial view (C), showing the intrauterine device (white arrow) in the inferior peritoneal cavity.

We present the case of a female in her early 30s who had undergone multiple prior uncomplicated pregnancies, all resulting in successful vaginal deliveries. She had no medical or surgical history. She presented to the emergency department (ED) with sharp, knifelike 10-out-of-10 left lower quadrant pain with associated nausea and vomiting. The pain was sudden in onset and started on the morning of her presentation to the ED. On initial presentation, she described the pain quality as “contractions” that radiated to the right lower back. The pain subsided on its own by the time of presentation (in the afternoon) with dull residual 6-out-of-10 achy pain in the area. There was no abnormal vaginal bleeding or abnormal discharge. She reported that her last menstrual period was on the day of presentation (the patient was currently menstruating). She denied dysuria or hematuria, as well as diarrhea, fever, or chills. She had a Mirena® (Bayer) IUD placed four years before her ED presentation. She had been sexually active, assuming protection from the IUD, since that time. However, she had no pregnancies. She was not using alternative methods of birth control. Notably, the patient had mild lower abdominal pain starting at least two to four months before ED presentation. The pain was worse with menstruation. The patient’s vitals were normal. On examination, she had very mild tenderness in the lower abdomen; otherwise, she was nontender and had no peritoneal signs. Her abdomen was soft and nondistended with no surgical scars. Complete blood count and comprehensive metabolic panel were unremarkable. Pregnancy test (serum human chorionic gonadotropin) was negative. A CT scan of the abdomen and pelvis with intravenous contrast showed a left-sided obstructing 5 mm ureterovesicular junction kidney stone with moderate hydroureteronephrosis proximally. It also showed a 3 mm non-obstructing stone in the left upper pole of the kidney. The scan also showed an IUD in the inferior peritoneal cavity with no free air and no ascites (Figure 1).

**Figure 2:**
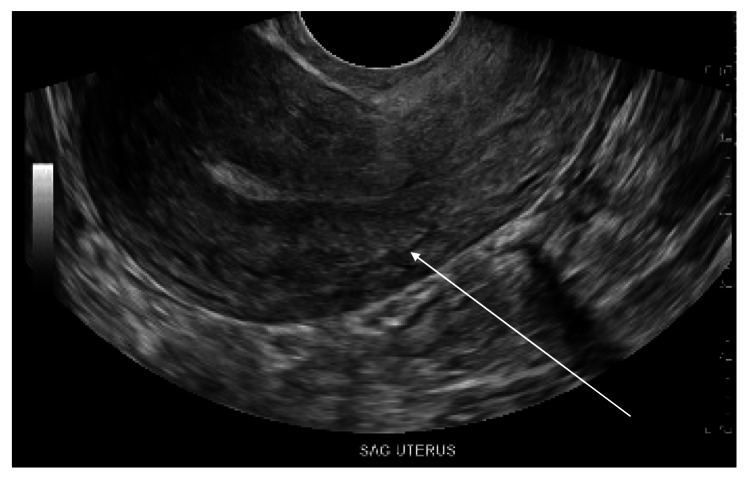
Transabdominal ultrasound demonstrating sagittal view of uterus with no intrauterine device seen (uterus denoted by the white arrow).

Transabdominal ultrasound of the uterus and adnexal structures showed no IUD within the uterus (which was anteverted), including the endometrial or endocervical canal (Figure 2).

**Figure 3:**
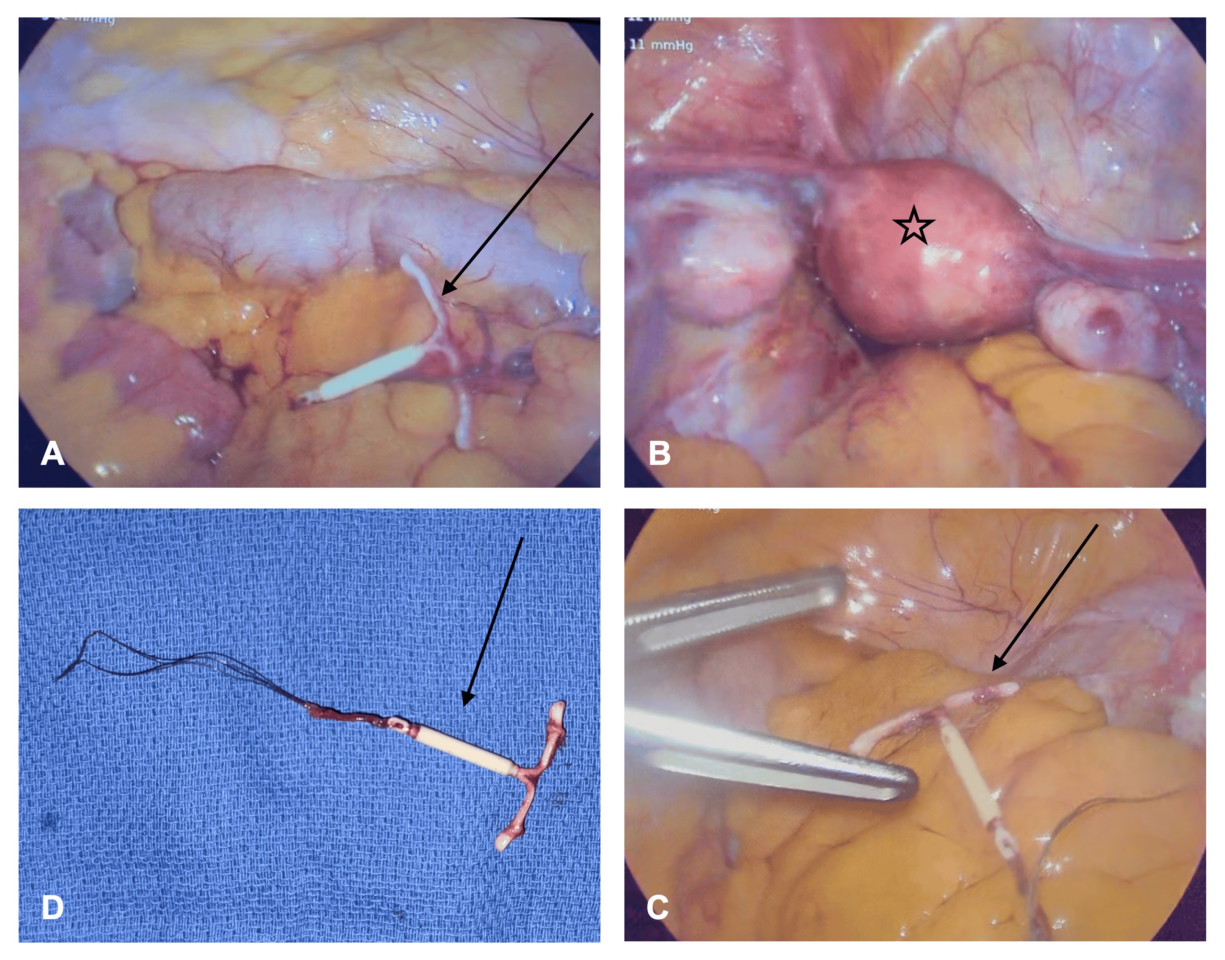
Intraoperative images demonstrating the intact intrauterine device (black arrow) with no associated adhesions in the peritoneal cavity (A, C). The uterus (black star) viewed with no defect or perforation (B). The intrauterine device with strings intact after retrieval (black arrow) (D).

Ultrasound was otherwise unremarkable. The urology service evaluated the patient and recommended medical management with tamsulosin for the stone. She was taken to the operating room with general surgery for a diagnostic laparoscopy. Intraoperatively, the IUD was visualized in the left paracolic gutter (Figure 3a). The uterus and fallopian tubes were inspected and found to be within normal limits, with no evidence of uterine perforation (Figure 3b). The gallbladder, stomach, cecum, ascending colon, appendix, rectum, sigmoid colon, and liver were also inspected and found to be within normal limits. The bowel was traced from the terminal ileum to the ligament of Treitz and was also within normal limits. The IUD was grasped by its strings and removed easily through the 12 mm port site (Figures 3c, 3d).

The IUD was free-floating in the peritoneal cavity and not encased in any adhesions or complicated by any abscess. The surgery was uncomplicated. The patient recovered well on the floor. She had an appropriate amount of incisional pain, tolerated her diet, ambulated, and had no other immediate postoperative complications. She was discharged with an outpatient follow-up scheduled. Notably, several months before presentation, she saw her obstetric and gynecology physician (OBGYN) in the clinic for a Papanicolaou smear, and they were unable to visualize the IUD strings. That was her first string check since the IUD was placed, according to the patient. The OBGYN recommended a transvaginal ultrasound, but the patient was lost to follow-up. The patient also reported that she had not followed up with her OBGYN between when the IUD was placed and the clinic visit; therefore, it is unknown how long the strings were not visible. This patient’s procedure report from the outside hospital was obtained. The IUD was placed by a certified nurse-midwife. The provider used a uterine sound, which sounded to 7 cm; a tenaculum was deemed unnecessary in this patient’s case. The IUD was inserted easily, and the patient tolerated the procedure well. All other appropriate steps were followed, and return precautions were provided to the patient. As previously mentioned, as the patient was lost to follow-up, a scheduled ultrasound was not taken.

## Discussion

As mentioned in the Introduction section, complications may occur at the time of IUD placement or later [3]. Complications include post-insertion bleeding, infection/pelvic inflammatory disease, expulsion, failure of contraception, malposition, and hormonal side effects. Uterine perforation, with an incidence of 0.1%, is an uncommon adverse effect that most commonly occurs at the time of insertion rather than delayed migration [3]. However, uterine spasms/contractions can cause delayed rupture with subsequent migration [4]. One study claimed that IUDs perforate the uterus invariably at insertion, and enough experience with inserting IUDs would eliminate the risk of perforation [5]. It is possible these IUDs migrate into the abdomen because of perforation or via transportation through the fallopian tubes [6]. The consensus is that the most common mechanism involves partial or complete perforation through the uterine wall at the time of insertion [7]. In cases of partial perforation, the device may remain embedded in the myometrium or subsequently migrate fully through the wall due to uterine contractions [7].

Previous case reports have reported ectopic IUDs. In these cases, a complication or involvement of another abdominopelvic structure is often present. Case reports have reported IUDs embedded in the appendix [8], penetrating the bladder [9,10], resulting in intra-abdominal abscess formation [11-13], perforating the small bowel [14], or the ureter [15]. One report showed it penetrating the sigmoid colon and as a cause of stricture of the sigmoid colon [16]. A case showed a copper T IUD in the extraperitoneal space of Retzius [17]. Another case showed a levonorgestrel IUD completely outside the uterus that caused adhesions with a resulting small bowel obstruction [18]. One case described a rare occurrence of a far-migrated intra-abdominal IUD detected incidentally 30 years after placement [4]. In this case, the patient refused surgery as she had been asymptomatic for so many years. The IUD was spiral-shaped on imaging and appeared to be a Lippes loop-shaped IUD [4]. The patient also had a pregnancy in that 30-year interval and a subsequent abortion in that case [4]. Although no symptoms or major complications were noted, because the IUD was not retrieved surgically, it was not known whether adhesions were present [4]. Copper IUDs are more likely to cause adhesions than non-copper IUDs [5]. Another case reported a T-shaped IUD that migrated into the left upper quadrant [6]. In this case, the patient was unable to recall which type of IUD was inserted, but intraoperative images showed a copper IUD [6]. This patient did recall an uncomfortable insertion process of the IUD 20 years prior [6]. She developed chronic abdominal pain and gave birth to two children during this 20-year interval [6]. The IUD was retrieved laparoscopically. A limited amount of filmy adhesive debris was found coating the copper coils in that case, but no severe adhesions or embedding was found [6]. A case of an IUD migrating into the mediastinum in a patient with a Bochdalek hernia has also been reported, with the appearance of a Lippes loop IUD on imaging [19].

The range of clinical presentation of migrated IUDs is variegated. It ranges from asymptomatic presentation to chronic abdominal or pelvic pain, to patients presenting with peritonitis in the setting of a perforated viscus or other major complications [6,12]. The timing of presentation from IUD placement varies widely as well, from recognition at the time of insertion to several decades afterwards, as described in multiple cases above [20]. All perforating IUDs into the peritoneal cavity should be removed, whether they present with symptoms or not, due to the risk of complications, including abscess, adhesions, fistula formation, and injury to the surrounding organs [21]. The laparoscopic approach is the current recommendation for the retrieval of all migrated peritoneal and myometrial IUDs, and laparoscopic removal is currently the most common approach to remove perforated IUDs [22]. Indications to utilize laparotomy include complex cases involving migrated IUDs perforating into the bowel or adjacent organs and significant adhesions [23]. As in other types of cases, surgeon experience and other factors may allow laparoscopic retrieval in even complex cases such as small bowel perforation from IUD migration [23]. Hysteroscopy alone or combined hysteroscopic/laparoscopic approach may be utilized to retrieve displaced IUDs still in the uterine cavity, depending on the degree of myometrial involvement [24].

## Conclusions

This case demonstrates that IUDs may rarely migrate into the peritoneal cavity and remain asymptomatic for several years without causing adhesions, perforations, or organ injury. In this case, the ectopic IUD was detected incidentally during evaluation of pain related to a kidney stone, and it was successfully retrieved laparoscopically without complication. This underscores the importance of maintaining vigilance in patients with absent IUD strings and ensuring appropriate imaging and follow-up after insertion. While IUDs remain a safe and highly effective form of contraception, this case emphasizes that early recognition of migration and prompt laparoscopic management can prevent potential long-term complications. Furthermore, the fact that the patient notably did not conceive during the four years following device placement raises the possibility of continued contraceptive effect despite intraperitoneal migration. However, as a single case, generalizability is limited. Larger cohorts are needed to assess contraceptive efficacy in migrated IUDs.

## References

[1] Britton LE, Alspaugh A, Greene MZ, McLemore MR (2020). CE: an evidence-based update on contraception. Am J Nurs.

[2] Madden T (2025). Intrauterine contraception: device types, characteristics, and counseling points. UpToDate.

[3] Lanzola EL, Auber M, Ketvertis K (2025). Intrauterine Device Placement and Removal. https://pubmed.ncbi.nlm.nih.gov/32491335/.

[4] Aydogdu O, Pulat H (2012). Asymptomatic far-migration of an intrauterine device into the abdominal cavity: a rare entity. Can Urol Assoc J.

[5] Soderstrom RM (1989). Trailing and treating the wandering IUD. Am J Gynecol Health.

[6] Sun CC, Chang CC, Yu MH (2008). Far-migrated intra-abdominal intrauterine device with abdominal pain. Taiwan J Obstet Gynecol.

[7] Rowlands S, Oloto E, Horwell DH (2016). Intrauterine devices and risk of uterine perforation: current perspectives. Open Access J Contracept.

[8] Sebai A, Elaifia R, Atri S, Hammami M, Haddad A, Kacem JM (2024). Intrauterine device migration resulting in acute appendicitis: a case report. Int J Surg Case Rep.

[9] Chen Z, Lv Z, Shi Y (2024). A case report of intrauterine device migration: uterine penetration and bladder involvement with secondary stones 3 years post-insertion. Int J Womens Health.

[10] Yu F, Chen M, Cao H, Yang G, Wang W, Wang Y (2024). Intrauterine device (IUD) migration completely into the abdominal cavity and half into the bladder to form a stone: a case report and mini-review. BMC Urol.

[11] Al-Darwish AS, Saffaf MK, Bawareth R, AlHawassi S (2025). Intraperitoneal migration of an intrauterine device: a case report. J Surg Case Rep.

[12] Benaguida H, Kiram H, Telmoudi EC, Ouafidi B, Benhessou M, Ennachit M, Elkarroumi M (2021). Intraperitoneal migration of an intrauterine device (IUD): a case report. Ann Med Surg (Lond).

[13] Ghoniem GM, Nguyen T (2024). Intravesical migration of intrauterine device: case report and literature review. Continence Rep.

[14] Chen CP, Hsu TC, Wang W (1998). Ileal penetration by a Multiload-Cu 375 intrauterine contraceptive device. A case report with review of the literature. Contraception.

[15] Karkin K, Vuruşkan E, Aydamirov M, Kaplan E, Aksay B, Gürlen G (2024). Hydronephrosis due to intraureteral migration of missed intrauterine device. Cureus.

[16] Brinth LS, Holte K, Andersen J (2007). [Penetrated IUD as the cause of stricture in the sigmoid colon]. Ugeskr Laeger.

[17] Georghiou P, Jaschevatzky OE, Grünstein S (1978). Extraperitoneal translocation of a Copper-T device into the space of Retzius. Acta Eur Fertil.

[18] Loveless A, Dhari A, Kilpatrick CC (2014). Perforated levonorgestrel-releasing intrauterine system resulting in small bowel obstruction: a case report. J Reprod Med.

[19] Amsriza FR, Fakhriani R (2021). Far-migration of an intrauterine device in the intrathoracic cavity: a rare case report. Clin Case Rep.

[20] van Grootheest K, Sachs B, Harrison-Woolrych M, Caduff-Janosa P, van Puijenbroek E (2011). Uterine perforation with the levonorgestrel-releasing intrauterine device: analysis of reports from four national pharmacovigilance centres. Drug Saf.

[21] Kho KA, Chamsy DJ (2014). Perforated intraperitoneal intrauterine contraceptive devices: diagnosis, management, and clinical outcomes. J Minim Invasive Gynecol.

[22] Santos AP, Wetzel C, Siddiqui Z, Harper DS (2017). Laparoscopic removal of migrated intrauterine device. BMJ Case Rep.

[23] Rahnemai-Azar AA, Apfel T, Naghshizadian R, Cosgrove JM, Farkas DT (2014). Laparoscopic removal of migrated intrauterine device embedded in intestine. JSLS.

[24] Situmorang H, Argy G, Putri A, Gunardi ER (2022). Intrauterine device translocation: case series and management algorithm. J Reprod Healthc Med.

